# Variations on a theme: Genomics of sex determination in the cichlid fish *Astatotilapia burtoni*

**DOI:** 10.1186/s12864-016-3178-0

**Published:** 2016-11-07

**Authors:** Astrid Böhne, Catherine A. Wilson, John H. Postlethwait, Walter Salzburger

**Affiliations:** 1 Zoological Institute, University of Basel, Vesalgasse 1, 4051 Basel, Switzerland; 2 Institute of Neuroscience, University of Oregon, Eugene, OR USA

**Keywords:** Sex determination, Cichlid, Wnt4, Sex chromosome, RAD sequencing

## Abstract

**Background:**

Sex chromosomes change more frequently in fish than in mammals or birds. However, certain chromosomes or genes are repeatedly used as sex determinants in different members of the teleostean lineage. East African cichlids are an enigmatic model system in evolutionary biology representing some of the most diverse extant vertebrate adaptive radiations. How sex is determined and if different sex-determining mechanisms contribute to speciation is unknown for almost all of the over 1,500 cichlid species of the Great Lakes. Here, we investigated the genetic basis of sex determination in a cichlid from Lake Tanganyika, *Astatotilapia burtoni*, a member of the most species-rich cichlid lineage, the haplochromines.

**Results:**

We used RAD-sequencing of crosses for two populations of *A. burtoni*, a lab strain and fish caught at the south of Lake Tanganyika. Using association mapping and comparative genomics, we confirmed male heterogamety in *A. burtoni* and identified different sex chromosomes (LG5 and LG18) in the two populations of the same species. LG5, the sex chromosome of the lab strain, is a fusion chromosome in *A. burtoni. Wnt4* is located on this chromosome, representing the best candidate identified so far for the master sex-determining gene in our lab strain of *A. burtoni*.

**Conclusions:**

Cichlids exemplify the high turnover rate of sex chromosomes in fish with two different chromosomes, LG5 and LG18, containing major sex-determining loci in the two populations of *A. burtoni* examined here. However, they also illustrate that particular chromosomes are more likely to be used as sex chromosomes. Chromosome 5 is such a chromosome, which has evolved several times as a sex chromosome, both in haplochromine cichlids from all Great Lakes and also in other teleost fishes.

**Electronic supplementary material:**

The online version of this article (doi:10.1186/s12864-016-3178-0) contains supplementary material, which is available to authorized users.

## Background

Sexual reproduction originated in the last common ancestor of eukaryotes and is almost ubiquitous in the animal kingdom [1]. Surprisingly for such an old trait, the initial triggers of sex determination (SD), the process driving the undifferentiated embryo towards a female or male phenotype, are not conserved. Classically, SD has been divided into genetic sex determination (GSD) and environmental sex determination (ESD, including e.g. temperature and the social environment). The best-studied form of GSD relies on a pair of sex chromosomes with one homolog carrying a master SD locus necessary for the development of one sex and when missing, leading to the development of the opposite sex.

The emergence of a novel SD gene, for example by gene duplication, transposition or allelic diversification, may initiate the formation of a proto-sex chromosome pair from autosomes (reviewed in [2]). Linkage to further genes impacting SD may then lead to the evolution of suppressed recombination, which extends the SD region, followed by the accumulation of deleterious mutations on the homolog with the SD gene due to enhanced genetic drift, background selection, Muller’s ratchet and genetic hitch-hiking (reviewed in [3]). The homolog carrying the SD locus may hence progressively degenerate with the expansion of the non-recombining segment facilitated by linkage disequilibrium and sexually antagonistic selection [4]. Such degenerated, heteromorphic sex chromosomes are exemplified by the mammalian Y chromosome or the avian W; in contrast to this, sex chromosomes in fish, if present at all, are often homomorphic and differentiation is not detectable by karyotyping [2]. Overall, fish show frequent turnover of sex-determining systems [5], which stands in sharp contrast to other vertebrate clades, especially to birds or mammals, where an entire class shares the same sex chromosomal system with sex being determined at fertilization.

So far, sex determination has been studied only in a small fraction of the over 30,000 described teleost species. Cichlid fishes with their outstanding adaptive radiations in the East African Great Lakes [6] offer the ideal evolutionary framework to study the emergence of SD systems. Early data on several species already indicate a high plasticity of SD systems, including male and female heterogamety as well as polygenic systems and even supernumerary B-chromosomes carrying SD loci [7–11]. African cichlids show a rather conserved karyotype allowing a direct comparison between species of chromosomes involved in SD [12]. In the mbuna cichlids from Lake Malawi (LM), an XX-XY system has been mapped to LG7, whereas a ZZ-ZW system in the same clade maps to LG5 [13]. Sex in two cichlid species from Lake Victoria (LV) has been mapped to a different region also on LG5 [8]. In the tilapiine lineage (basal to the lake radiations), which feature male and female heterogametic systems with additional ESD to some extent, sex-associated chromosomes include LGs 1, 3 and 23 [14, 15].

Here we set out to investigate the genetic basis of sex determination in *Astatotilapia burtoni*, a cichlid that inhabits Lake Tanganyika (LT) and its tributaries. *A. burtoni* has become an important fish model system especially in behavioral research and neurobiology but also in genetics and genomics [16–24]. This sexually dimorphic species, in which males are larger and more brightly colored than females, belongs to the most derived and species-rich lineage of East African cichlids, the haplochromines, which is the lineage found in all three Great Lakes [25]. We previously showed that our laboratory population of this species has an XX-XY sex chromosomal system [10].

Genomic analyses based on next generation sequencing data have rapidly increased the identification of sex chromosomal systems in different taxa (e.g., [26, 27]). To further disentangle the genetics underlying sex determination, we applied RAD-tag population genomics to offspring of two *A. burtoni* crosses. Curiously, results showed different sex-associated chromosomes in each of the two populations.

## Methods

### Crosses

Fish were kept at the aquarium facilities of the Zoological Institute of the University of Basel under standard conditions [23]. A male-female pair of the reference lab strain of *A. burtoni* (inbred line) as well as a male-female pair of *A. burtoni* caught at the estuary of the Kalambo river in LT at Chipwa village (Additional file 1: Figure S1) were crossed. Offspring of each cross (two broods each for lab strain and the Chipwa population) were raised until sexing was possible based on the appearance of sex-specific color patterns (~4 months post hatching; near equal sex-ratios, yellow-orange color markings on the anal fin of male fish, so-called egg-spots, and typical male body coloration were clearly visible). Fish were then fin-clipped and kept for an additional three months to confirm sexing by visual gonad inspection after dissection.

The analysis included the two parents of each cross as well as 28 sons and 27 daughters for the lab strain and 20 sons and 20 daughters for the Chipwa cross.

### RAD-sequencing

DNA of each individual was extracted from caudal fin tissue with a Qiagen QIAamp DNA mini kit and quantified using a Qubit Fluorometer. RAD-sequencing library preparation was done at the University of Oregon, Eugene, following the method described in [28]. In brief, DNA of each individual fish (500 ng in 50 μl) was digested with SbfI (high-fidelity enzyme, New England Biolabs no. R3642S). Each individual DNA was labeled with a 5-nucleotide barcode adapter ligated to each restriction site during library preparation. Libraries were sequenced on an Illumina HiSeq 2500 using the 100 bp-single-end mode. The software package Stacks (version 1.20, [29, 30]) was used to organize reads into loci and to identify sex-related polymorphisms (SNPs within the loci) using the “RAD-sex” approach [31] as described in the following. First, reads were sorted and filtered using “process_radtags” of Stacks. Next, we ran the reference-free “denovomap.pl” pipeline of Stacks for the lab strain and the Chipwa offspring separately, to generate a catalog of loci present in each population (settings: -m minimum stack depth 0, -M differences when merging stacks into loci 4, and –n differences between loci when building the catalog 2). To identify SNPs associated with sex, we defined males and females each as one population. We ran the “populations” program of Stacks, which calculates *F*_ST_ values and associated statistics for pairwise comparisons (here males and females) [29]. We ran “populations” separately for the lab strain and wild fish, each with defining minimum number of individuals required for a locus in each population as 1 (-p 1) and minimum stack depth required for individuals at a locus as 3 (-m 3) excluding over-merged loci (-B blacklisted markers, generated with a custom python script [31]). We further included a *p*-value correction to *F*_ST_ values (-f *p*_value). Resulting SNPs with corresponding statistics including *F*_ST_ and associated *p*-values of Fisher’s Exact Test implemented in “populations” [29], were exported in text format for further inspection.

### Coverage analysis

To identify potential sex-associated RAD loci by differences in coverage in male and female genomes, we used the sequencing reads of the two fathers to construct reference loci with ustacks (-m 3, -M 4). These loci were extracted from the Stacks tsv output and transformed into fasta format. The loci were indexed using Bowtie2 (version 2.2.2, [32]) and then reads of each offspring were aligned against this reference dataset in sensitive local alignment mode of Bowtie2. Lab strain offspring were aligned against the lab strain father reference, Chipwa offspring against the Chipwa father reference. Alignments of each individual in SAM format were sorted, indexed and converted to BAM format using SAMtools (version 1.2, [33]). Next, sequence coverage per file was extracted from BAM files with BEDTools (version 2.25, [34]) using the genomeCoverageBed function. A custom perl script was used to calculate mean coverage per RAD locus for each male and female offspring separately. Subsequently, the mean of all means was calculated for each mapped locus for sons and daughters. Loci with a mean coverage below 1 (spurious reads) and above 500 (likely repeat motifs) were filtered out in R. From the remaining loci, we extracted those with a coverage below 2 in females (spurious reads or reads from wrongly sexed individuals) and above 5 in males as male-only coverage loci. For the lab strain, the resulting 41 loci were manually inspected in the original Stacks catalog for presence and absence in males and females. For loci present in at least 10 males, PCR primers were constructed at the very beginning and end of the 100 bp locus and amplified on male and female DNA by PCR using RedTaq (Sigma Aldrich, Primers and PCR condition see Additional file 2: Table S1). We also inspected loci with a coverage ratio in daughters/sons over 1.5. We mapped the father’s loci on the Nile tilapia genome (standalone BLAST+/2.2.31, blastn, e-value cutoff of 0.001) and recovered LG placement information from the best hit of each locus. We then tested for an over representation of male-only and female-biased loci on LG5 in the lab strain and LG18 in the wild fish (two-tailed Fisher’s exact test).

### Comparative genomics

The sequences of RAD loci of interest were compared to the genomes of *A. burtoni* and *O. niloticus* (https://blast.ncbi.nlm.nih.gov/Blast.cgi, blastn, default settings) to determine their chromosomal locations. Contig and gene positions were derived from NCBI map viewer. To compare sex-determining regions of different cichlid species on LG5, sequence information was retrieved from (Figure S2 in [7]) and the NCBI SNP archive for [35]. The obtained sequences were blasted against the Nile tilapia genome to locate them.

### Gene expression

To investigate male and female expression of genes located on the potential sex chromosome of *A. burtoni*, filtered RNA-sequencing reads of testis and ovary tissue from [22] were mapped against a non-redundant mRNA dataset of *O. niloticus* retrieved from the RefSeq RNA GBF file accession number GCF_000188235.2 keeping only the longest isoform for each gene. The reference was indexed with Bowtie2 (version 2.2.2, [32]) and reads were then mapped in local mode (settings --local -D 20 -R 3 -N 1 -L 20 -i S,1,0.50). Resulting alignments were reported in SAM format and sorted, indexed and transformed into count tables (number of mapped reads per transcript per sample) using SAMtools (version 1.2, [33]). Count data were imported in R and differences in gene expression were analyzed using the Bioconductor edgeR package (version 3.12, [36]) with the implemented GLM approach with quasi-likelihood F-tests.

### Gene ontology

To investigate GO representation of genes located on Nile tilapia LGs 5, 14 and 18, gene sequences were exported from NCBI, loaded into blast2GO, blasted against nr, mapped and annotated with default settings.

## Results

### Polymorphic sites support male heterogametic systems in *A. burtoni* lab strain and Chipwa

For the inbred lab strain cross, we obtained 5,358 polymorphic sites in males and females (located in 2,969 RAD loci). For the wild type Chipwa cross, we obtained 24,185 polymorphic sites (located in 11,633 RAD loci). We inspected the 100 most significant sites for each cross (Additional file 3: Table S2 and Additional file 4: Table S3, ordered by Stacks Fisher’s *p*-value). In the lab strain, at 52 of these SNPs, father and sons are heterozygous whereas daughters and the mother are homozygous; meaning that only sons inherit specific alleles from the father, as expected from an XX-XY sex-determining system. At most of the remaining loci, mother and father were both heterozygous. In the Chipwa offspring, we found 19 SNPs among the 100 most significant ones in which inheritance patterns also support an XX-XY system. Again, at the other loci, either both parents are heterozygous or the loci represent repetitive motifs.

### A fused chromosome is the sex chromosome in the *A. burtoni* lab strain

For *A. burtoni*, a rather fragmented reference genome from a female individual is available, which frustrates long-range conserved synteny analyses that are possible with a ‘chromonome’, a chromosome-length genome assembly [37]. Fragmented assemblies also present the problem that small scaffolds are less likely to contain genetic polymorphisms that can be readily assayed without whole genome sequences from many tens of individuals. The only currently available cichlid chromonome assembly is from the Nile tilapia (*Oreochromis niloticus*), a member of a lineage basal to the cichlids of the Great Lakes. Chromosomal organization is largely conserved among cichlids, although *A. burtoni* has fewer chromosomes (*n* = 20) compared to *O. niloticus* (*n* = 22) due to two chromosome fusions [38]. One of the fusions comprises *O. niloticus* LG15 and LG19 [8]. In the second fused chromosome, one of the partners corresponds to LG5 in *O. niloticus* [38], but the second remains to be identified.

**Fig. 1:**
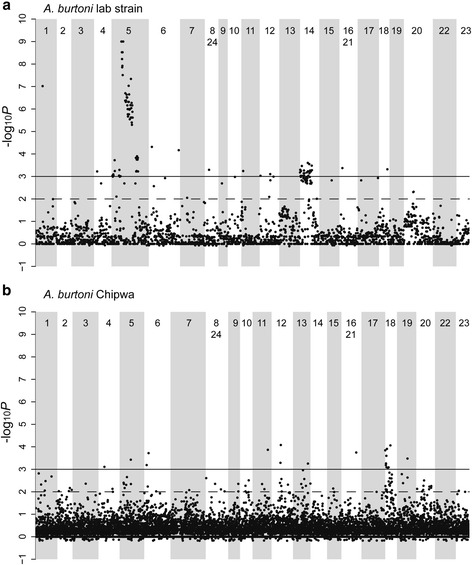
Association of chromosomes with phenotypic sex in **a** *A. burtoni* lab strain and **b** Chipwa wild-caught fish. The plots show the –log10 *p*-values of genotypes associated with sex plotted against the Nile tilapia chromosomes used as reference genome. Odd-numbered chromosomes have a *white background* while even-numbered chromosomes have a *grey background*. The *solid line* corresponds to a *p*-value cut off of 0.001, the *dashed line* to a *p*-value of 0.01

We located all RAD loci of interest (2,969 for the lab strain and 11,633 of the Chipwa fish) in the Nile tilapia genome (Fig. 1) In the lab strain, we detected a strong association between phenotypic sex and the fused chromosome 5 and to some extent chromosome 14 (sharp peak on LG5, weaker peak on LG14 in Fig. 1a). In the Chipwa fish, we did not find the same association, but a peak on LG18 (Fig. 1b). In the lab strain, the highest density of the most significantly associated SNPs corresponds to a ~14 MB region on LG5, located between 6.8 and 20.3 MB, referred to as SD region (Fig. 3, blue box).

An examination of the parental origin and scaffold location of sex-associated SNPs in the lab strain fish showed that the majority of SNPs (48 out of 52) that show clear transmission of paternal alleles to only sons, are on LG5, while the remaining 5 SNPs mapped to unplaced scaffolds (Additional file 3: Table S2). These loci could either be minor sex modifiers not placed on the sex chromosomes, be wrongly assembled in the Nile tilapia genome or be rearranged in the *A. burtoni* genome.

In the Chipwa fish, 12 out of the 19 SNPs that show inheritance patterns supportive of an XX-XY system mapped to LG18 spanning ~16 MB, the remaining ones mapped to LGs 1, 20, 12, 8-24 and to unplaced scaffolds (Additional file 4: Table S3).

### Confirmation of the sex chromosome in *A. burtoni* by sequence coverage analysis

**Fig. 2:**
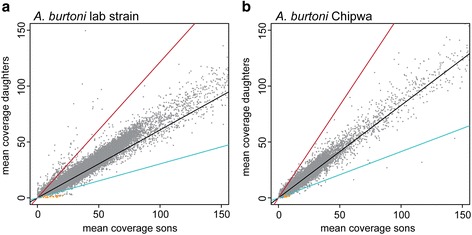
Sequence coverage of paternal RAD loci in sons and daughters of **a** lab strain and **b** Chipwa wild-caught fish. Autosomal loci should show equal coverage in both, males and females (*along black line*), whereas X-linked loci should have twice the coverage in females compared to males (*red line*, the expectation for W-linked loci in a ZZ-ZW system is shown as *blue line*, corresponding to double coverage in males; lines are adjusted for differences in sample size and mean coverage). Most loci show similar coverage in sons and daughters. Note that some loci have coverage only in sons, especially in the lab strain, and are depicted in *orange*

Despite the fact that sex chromosomes usually have high repeat content, some RAD-tags might be predicted to be only on the Y-chromosome and some only on the X-chromosome, being located in the non-recombining part of the sex chromosome. For such loci, differences in sequence coverage in males and females enabled us to perform a second – and independent – approach to identify ancestral LGs in the *A. burtoni* sex chromosome. In an XX-XY sex-determining system, sequences present on the Y but absent from the X chromosome should have sequence coverage only in males, whereas sequences in an X-limited region should be twice as frequent in XX females compared to XY males. In contrast, sequences in pseudo-autosomal regions (PARs), should have the same coverage in males and females like autosomal region. This copy number variation strategy has previously been used to identify non-recombining regions on fish sex chromosomes [39, 40]. To identify male-limited loci, we built a RAD locus catalog from the father as a reference (instead of using the female reference genome, which would lack Y-limited sites) and mapped reads of each son and each daughter to this reference. We then calculated mean coverage for all sites in these loci (Fig. 2a). We detected 41 loci with male-only coverage (orange dots on Fig. 2), with an over representation of such loci on LG5 in the Nile tilapia genome (*p*-value < 2.2e-16; for further RADtag distribution see Additional file 2: Table S1). We inspected all male-only coverage loci in the initial Stacks catalog and constructed PCR-primer pairs for the 19 loci present in at least 10 sons to test their presence/absence by PCR on male and female DNA. Two loci indeed amplified only from DNA from sons of the cross. However, when extended to more individuals of our lab strain, the two markers occasionally amplified bands of the expected size also in females. This result could indicate either sex reversed individuals or recombination events between the sex-linked markers and the sex-determining locus.

Concerning loci with higher coverage in daughters than sons (ratio daughter/son >1.5 with an initial minimum coverage >2 in females to exclude false positives caused by too low sequencing depth), we also found an over representation on LG5 (*p*-value = 7.894e-06; 201 total loci, 128 placed on LGs, 22 on LG5) but also on LG1 (*p*-value = 5.3e-11, 27 loci).

In the Chipwa cross, again, we detected fewer loci both with male-only and daughter/son >1.5 coverage than in the lab strain cross (Fig. 2b and Additional file 5: Table S4). Nevertheless, LG18 has an over representation of these loci (29 loci with male-only coverage, 27 loci placed on LGs, 6 on LG18, *p*-value = 0.0002; 102 loci with daughter/son >1.5, 78 placed on LGs, 14 on LG1, *p*-value = 2.361e-07).

### Candidate genes on *A. burtoni* sex chromosomes

Some genes have repeatedly evolved as master sex determiners in tetrapods, including *Sox* and *DM* domain factors and, especially in fish, also members of the TGF-beta signaling cascade (reviewed in [5]). These “usual suspects” [41] derived by duplication or allelic diversification from genes with a known function in sex differentiation or gonad development. However, sex-determining genes can also be “newcomers” [41] as the *sdY* gene in the rainbow trout, which shows no similarity to any described gene with a function in sexual development [42].

In the Chipwa fish, the region with SNPs associated with sex on LG18 spans ~16 MB reaching from position 3.9 MB to 19 MB and containing 731 genes. None of these genes has been described as a sex-determining gene before.

In the lab strain, the sex-associated region on LG5 spans the first 28 MB of this 37 MB-long chromosome. This region has 966 annotated genes in Nile tilapia. The region on LG14 spans the first 15 MB and includes 397 annotated genes.

**Fig. 3:**
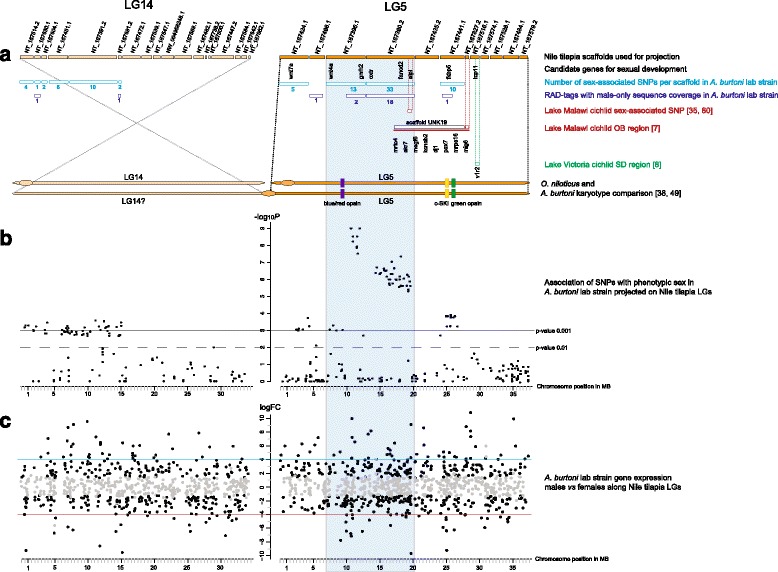
Comparative genomics of LG5 in haplochromine cichlids of Lakes Tanganyika, Malawi and Victoria. In *A. burtoni*, LG5 has a centric fusion to another chromosome upstream of the blue/red opsin genes [38], probably LG14, here shown on the left site. **a** LG5 and LG14 show sex association in *A. burtoni* lab strain. SNP inheritance patterns on LG5 support an XX-XY system on this chromosome in *A. burtoni* lab strain. LG5 carries a ZZ-ZW SD locus in Lake Malawi cichlids as well as a SD locus in Lake Victoria cichlids. *Light blue* numbers and boxes correspond to sex-associated SNPs in this study. Gene candidates for sex determination on LG5 are depicted below the corresponding scaffolds of the Nile tilapia. *Dark blue *boxes and numbers indicate location of male-specific loci. Sex-determining regions of other cichlids are indicated for Lake Malawi in *red* and Lake Victoria in *green*. **b** The plots show the –log10 *p*-values of genotypes associated with sex plotted against the Nile tilapia chromosomes LG5 and LG14 as higher resolution of Fig. 1. **c** Gene expression does not show a particular accumulation of sex-biased genes on LG14 or LG5 in *A. burtoni* lab strain (*black dots* correspond to significantly differentially expressed genes, *grey dots* correspond to not differentially expressed genes; values above 0 correspond to overexpression in males, values below 0 to overexpression in females, see Additional file 8: Table S6 for details). The *blue box* highlights the region with the highest density of Y-linked loci and sex-associated SNPs with the most significant *p*-values, referred to as SD region

LG5 is also sex-associated in other cichlids. In addition, it contains several genes that have already been implicated in sexual development and reproduction, including two secreted signaling proteins *wnt7a* and *wnt4a*, which are known to control cell fate choices [43]. *Wnt4* is an especially likely candidate in sex determination and plays an important role in gonad development in mammals [44–46]. It is located at the beginning of the SD region of *A. burtoni* lab strain (Fig. 3a). Two other genes on LG5 play a role in hormonal regulation of reproduction, including *gnrh2* (*gonadotropin-releasing hormone 2*), and *oxtr* (the *oxytocin receptor*). The final candidate gene is *tcp11* (*T-complex protein 11 homolog*), which might play a role in sperm function and fertility ([47] and references therein).

To characterize further candidate genes in chromosomal regions with sex-associated SNPs, we examined genes placed in the three regions on LG5, 14 and 18 for their gene ontology (GO) terms searching for categories related to sexual development and reproduction (“sex determination GO:0007530, sex differentiation GO:0007548, developmental process involved in reproduction GO:0003006, reproductive process GO:0022414 and reproduction GO:0000003), allowing a hunt for the “usual suspects” (Additional file 6: Table S5). We only identified two genes in this way, *alpl* (*alkaline phosphatase*) with a GO annotation GO:0003006, and *fancd2* (*fanconi anemia complementation group d2*) with GO:0000003, both located on LG5 (see Fig. 3 for location of all candidate genes). The FANC gene family has especially been investigated for its role in zebrafish sex determination [48].

### Male and female gene expression in *A. burtoni* lab strain

Sex chromosomes often show a sex-specific gene content, which can also be reflected in gene expression. For the *A. burtoni* lab strain, male and female expression data are available from [22], unfortunately no expression data are currently available for the Chipwa population.

We here investigated gene expression along LG14 and LG5 using adult male and female gonad RNA sequencing reads from [22]. Sex-biased genes were not accumulated on LG5 or LG14 and gene expression did not show a particular pattern on these or any other chromosomes (Fig. 3c and Additional file 7: Figure S2 for all other chromosomes). We inspected the genes that showed the strongest sex-bias on these two chromosomes (logFC >4, genes above blue line in Fig. 3c are highly male-biased, genes below the red line highly female biased, Additional file 8: Table S6), and investigated gene expression of the above mentioned candidate genes (Additional file 8: Table S6). From the candidates, *wnt4a* is the only one that shows over expression in male gonads, while *wnt7a*, *fkbp5* and *tcp11* are overexpressed in females. *Gnrh2*, *otxr*, *fancd2*, and *alpl* are not differentially expressed. Note however, that downstream of *wnt4a* several genes show strong sex biased expression (Fig. 3. blue box, Additional file 8: Table S6).

The genes that are most differentially expressed and show the highest expression in males on LG5 include another member of the wnt pathway (*calcoco1*), several transcription factors, and genes involved in cell migration/motility. The gene with the strongest male-bias is *sycp1*, synaptonemal complex protein 1 (at ~29 MB of LG5 in the Nile tilapia, Fig. 3c), reflecting the many meioses occurring in testes. Genes with a strong female bias on LG5 also include several transcription factors. The gene with the strongest female-bias is *histone H100-like* (at ~20 MB of LG5 in the Nile tilapia, Fig. 3c).

## Discussion

### The sex chromosome in *A. burtoni* lab strain is a fused chromosome

The SD region that we identified in *A. burtoni* lab strain is located between position 6.5 MB and 20 MB on LG5 of the Nile tilapia. In Nile tilapia, the telocentric centromere is placed close to this region [38, 49]. The homologous chromosome to LG5 in *A. burtoni* is a large, metacentric chromosome, likely the result of a centric fusion of two subtelocentric/acrocentric chromosomes [38]. So far only LG5 was known to be involved in this fusion [38]. In the lab strain fish, we detected sex-association with a second Nile tilapia chromosome, LG14 (smaller peak in Fig. 1a), and hence suggest that this chromosome is the one fused to LG5 in *A. burtoni*. Our association pattern with sex on both Nile tilapia chromosomes LG5 and LG14 in vicinity to the telocentric centromers, supports a centric fusion as previously proposed for this chromosome in *A. burtoni* (Fig. 3a, [38]). Numerous neo-sex chromosomes have been characterized in fish, most often generated by Y-autosome fusion [50, 51]. Such fusions are easily recognizable because they cause an odd number of chromosomes in a diploid karyotype of one sex and might be implicated with speciation (e.g. [50, 52, 53]). In *A. burtoni*, males and females have the same karyotype of 2n = 40 ([12], personal communication with Cesar Martins) indicating that chromosome fusions were not causing the formation of neo-sex chromosomes. Most other African cichlids show a 2n = 44 karyotype [12]. However, data are not sufficient to date the two fusions in *A. burtoni* nor to correlate them with the emergence of SD loci on LG5.

### Varying sex determination systems in two populations of *A. burtoni*

Usage of different sex chromosomes in closely related sister species has previously been described for the fish genus *Oryzias* (reviewed in [54]) as well as in populations of sticklebacks [55]. Our laboratory population of *A. burtoni* provided strong genetic evidence for an XX-XY sex-determining system located on the fused chromosome LG5/LG14 ([10] and this study). In addition, Roberts et al (DOI 10.1186/s12864-016-3177-1) found a sex-asociated locus on LG13 in some families of their laboratory population that we did not detect. In offspring of Chipwa fish, we have support for yet another XX-XY locus on LG18 although the genetic signal is somewhat weaker than the one detected in the lab strain (Fig. 1). The wild-caught fish studied here are offspring from a couple caught at the Chipwa village at the estuary of the Kalambo river, which flows into LT (Additional file 1: Figure S1). The lab strain was originally derived from another population, which is closely related to current populations from the northern part of the lake, about 600 km away from Chipwa (Pauquet et al. in preparation). Note that the *A. burtoni* genome was also derived from a female of a laboratory strain, the one of the Hofmann lab (inbred line for ~60 generations [20]), which belongs to the same clade as the lab strain investigated here. Hence, the two studied crosses belong to geographically as well as genetically distantly related populations (Pauquet et al. in preparation), which could account for differences in their sex chromosomal systems.

In zebrafish, domesticated strains have lost the natural sex-determining system probably as a by-product of selection under laboratory conditions [31]. Under laboratory rearing, fish are often selected for early maturation and breeding as well as fast growth and probably also balanced sex ratios. A second possible explanation for differences in the sex-determining system and also their degree of differentiation in the two investigated *A. burtoni* populations is that the sex-determining system in our *A. burtoni* lab strain has been co-selected in such a process.

### The sex-determining regions of *A. burtoni*

The SD regions of *A. burtoni* span ~14 MB in the lab strain (blue box in Fig. 3) and ~16 MB in the wild-caught fish. In the lab strain, sex-associated SNPs can even be seen along a region of much longer size (~28 MB on LG5 and ~14 MB on LG14). The association signal of sex determination in the wild-caught Chipwa population is weaker than the one in the lab strain, which might be explained by a generally higher level of heterozygosity in wild compared to inbred fish. It could also be that the sex chromosomes of the Chipwa population are even younger or less differentiated than the one of the lab strain/northern populations or that sex determination is polygenic.

The same RAD-sequencing method detected comparable regions of sex-association of 18.2 MB around the SD locus in the medaka [31] in which the Y-chromosome specific part is only 250 kb long and the SD system is about 5–10 million years old. ([56] and references therein). In the three-spined stickleback with ~10my old sex chromosomes, sequence differentiation between X and Y as reflected by elevated nucleotide divergence, accumulation of Y-specific alleles as well as rearrangements covers a region of 16.4 Mb (over 80 % of the X-chromosome length), however being much larger than the actual SD locus [57, 58]. We hence expect that the effective locus responsible for SD in *A. burtoni* will be smaller than the 14 and 16 MB, respectively. Regions around loci under sexually-antagonistic selection (as the color and vision genes on LG5, Fig. 3) can show elevated divergence between X and Y and linkage to the SD locus can make the divergence peak much broader than without this linkage (e.g., on an autosome) [59]. The rather large regions of sex-association that we detect could reflect this pattern. A possible explanation for increased heteromorphism on (young) sex chromosomes along a rather large region are structural rearrangements such as deletions or inversions accompanied by a reduction in recombination [57]. The causality or order of these events is however debated ([60] and references therein).

Lack of data from more closely related species as well as the sequencing resolution makes dating of the sex chromosomal system as well as defining the SD locus in *A. burtoni* difficult. Comparing wild-caught fish and lab fish, artificial selection under laboratory conditions could have slightly speeded up the process of sex chromosome differentiation in the lab strain as well as reduced the level of heterozygosity accounting for the broader distribution of sex-associated SNPs as well as the higher significance levels of association (visible when comparing the Chipwa fish and lab fish, Fig. 1a and b).

Sequence coverage comparison of males and females confirms sex-association with LG5 in the lab strain and LG18 in the wild-caught fish. However, it does not indicate sequence differentiation along those chromosomes as, for example, it did in sticklebacks [39]. PCR of potential Y-markers identified in the coverage analysis, did not detect complete sex linkage in individuals of the lab strain that were not members of the investigated cross. Also, these markers are present in the *A. burtoni* reference genome, which is derived from a female individual. This finding suggests that the regions we identified are still quite similar between X and Y and recombine over most their length, which are usually thought to be properties of rather young sex chromosomes.

Other explanations are that sex reversal [23] or intersex individuals can occur in our lab strain (we occasionally observed individuals that had a male phenotype but lay eggs as well as one intersex individual with a mixed gonad, A. Böhne and B. Egger, personal observations). Sex reversal can counteract Muller’s ratchet by purging deleterious mutations through occasional recombination in XY females, which results in rejuvenating sex chromosomes, preventing them from strong differentiation and decay and thus accounting for homomorphic sex chromosomes [60, 61]. It could also be that genetic sex determination is incomplete in *A. burtoni* and can be impacted by environmental factors.

Differentiated (heteromorphic) sex chromosomes often show a non-random distribution of sex-biased genes [62–65]. We did not, however, see any sex-bias in gene expression along LGs 5 and 14, a result that also indicates not yet specialized, young chromosomes. We previously showed that the ancient LG1 sex chromosome contains an over-representation of genes commonly expressed in a female-biased manner in several LT cichlids [22].

Overall, our method identified the sex-determining regions of two *A. burtoni* populations but it has not yet identified the non-recombining Y-limited regions and suggests that sex chromosomes in both populations are rather young and undifferentiated. Because our RAD marker density is estimated to average about one cutting site every ~30 kb, it could miss a small Y-limited region. This situation was indeed the case in the medaka [31], where the major SD gene is on an unassembled scaffold with no *SbfI* restriction site.

### “Limited options” – *wnt4* is a promising candidate for sex determination in *A. burtoni*

Except for birds or mammals, sex-determining genes do not tend to be strongly conserved within vertebrates. Even novel genes not related to any known sex-differentiating gene can become a master SD locus [42]. Still, it seems, that a pool of genes referred to as “usual suspects” [41] or “limited options” [66] has repeatedly evolved as master SD genes, especially in fish.

Besides *sdY*, the novel gene in rainbow trout [42], the described teleost master SD genes either belong to the DM domain transcription factor family (*dmY*/*dmrt1bY* in the medaka *Oryzias latipes* [67, 68], and *Oryzias curvinotus* [69], *dmrt1* in Chinese tongue sole [40]), the Sox family (*sox3Y* in *Oryzias dancena* [70]) or the TGFbeta signaling pathway (*gsdfY* in *Oryzias luzonensis* [71] and sablefish [72], *amhY* in Patagonian pejerrey [73] and *amhr2Y* in Fugu [74]).

Also belonging to this pool of usual suspects is *wnt4*, a key gene of gonad development and sex determination in mammals [44], which is located at the beginning of the SD region of the *A. burtoni* lab strain (Fig. 3a) and presents our prime candidate for sex determination. Cichlids, like other teleosts, possess two copies of *wnt4* as they do for most of their genes as the result of a whole genome duplication at the origin of the teleostean lineage [75–77]. In a previous study, we showed that the expression profile of *wnt4b* (located on LG11) strongly resembles that of a male SD gene with an early expression peak in developing male gonads [10]. However, we did not detect any sequence difference between male and female individuals. In that study, we also profiled the expression of *wnt4a*, the gene copy located on cichlid LG5 (figure 3 in [10]). Within the time window studied, *wnt4a* did not show a peak in gonadal expression as sharp as its ohnolog *wnt4b*. Still, *wnt4a* expression showed a reduction in expression level throughout the experiment and a maximal expression level at the same time point as *wnt4b*. In addition, we detected here over-expression of *wnt4a* in adult testes. In olive flounder (*Paralichthys olivaceus*), overexpression of *wnt4a* in the developing male gonad has also been described [78]. Taken together, these findings support a possible function in SD or gonad development for *wnt4a* in *A. burtoni*.

### Comparative genomics of LG5 in cichlids

The concept of ‘limited options’ appears to apply not only to genes, but also to entire chromosomes [66, 79]. LG5 seems to be one such chromosome in teleosts. Different studies have identified LG5 as a sex chromosome in cichlids, including species from LM and LV, all belonging to the haplochromine lineage, which also contains *A. burtoni*.

A sex-associated SNP has been identified in LM cichlids with a ZZ-ZW sex-determining system in the same region on LG5 (at ~19.5 MB, [35, 80]). This ZZ-ZW system is coupled to the orange blotch (OB) genetic determinant in LM cichlids, located on LG5, with *pax7* as the responsible gene [7]. Of the OB region [7], only *mig6* is located on LG5, at 28 MB of the Nile tilapia, further downstream of the sex-SNP identified by [35]. The rest of the OB region is on the unassembled scaffold UNK19 of the Nile tilapia, which itself shows conserved chromosomal linkage in several fish genomes, including zebrafish, medaka, stickleback and the two pufferfish *Fugu* and *Tetraodon*, suggesting its placement on cichlid LG5 [7]. Note that in the *A. burtoni* lab strain, one of the significantly sex-associated SNPs supporting an XX-XY system is located on UNK19 (Additional file 3: Table S2), in agreement with linkage to LG5.

The third SD region on LG5 was identified in LV cichlids, located close to the pheromone receptor *v1r2* (figure 4 in [8])*,* at position ~30 MB on LG5, not overlapping with the SD regions in LM or LT. It is not clear whether this sex-determining system is chromosomally XX-XY or ZZ-ZW.

Some chromosomes are more often and repeatedly used as sex chromosomes than others [66, 79]. The preferential co-option and re-usage of a specific chromosome as sex chromosome could be associated with particular gene functions or sexual antagonistic effects of genes those chromosomes carry [81]. LG5 of cichlids seems to be one such chromosome. It has (most likely) independently been used as a sex chromosome in cichlids of the three Great Lakes possessing different heterogametic statuses. The SD region in LV cichlids does not overlap with the one in LM or LT cichlids. The XX-XY system on LG7 in LM is ancestral in this cichlid lineage [13] dating the emergence of the ZZ-ZW locus on LG5 after the split from LT cichlids, arguing also for independent evolution of LG5 as sex chromosome in cichlids of all lakes. However, the repeated co-option of LG5 as sex chromosome could still represent the reuse of a shared ancestral polymorphism responsible for SD in cichlids from the different lakes.

The evolution of this chromosome as sex chromosome is not confined to cichlids but chromosomes homologous to cichlid LG5 repeatedly evolved as sex chromosomes in other teleosts. Cichlid LG5 corresponds to TEL3 [49], the reconstructed ancestral teleost chromosome 3 [82], which is ancestral to the sex chromosomes LG12 in Ninespine stickleback, LG19 in Tiger pufferfish and LG5 in *Oryzias hubbsi* (reviewed in [66]). This repeated usage of descendants of TEL3 as a sex chromosome could be linked to the presence of sexually antagonistic genes on TEL3. On fish sex chromosomes, sexually antagonistic genes are often involved in pigmentation (table 2 in [83]). The homologs of TEL3 carry several pigmentation genes as well as some of the color vision opsin genes ([38], table 2 in [66]), making this chromosome one of the “limited options” that could evolutionarily be favored as sex chromosome due to location of sexually antagonistic loci on it and their linkage to SD genes.

## Conclusion

Although sex chromosomes are not conserved among fish species, particular chromosomes seem to be more likely to evolve as sex chromosomes than others. Cichlids, and especially the haplochromine lineage, are a perfect system to investigate sex chromosomes of different evolutionary ages or stages. Furthermore, they offer the possibility to study transitions between different sex chromosomal loci as well as their linkage to sexually antagonistic and novel key traits and eventually their contribution to speciation. Whole genome sequencing of different *A. burtoni* strains and populations might facilitate the identification of Y-chromosomal regions and shed light on the origin and potential turnover of sex chromosomes in haplochromines as well as help in the identification of novel genes located on the sex chromosomes although assembly of repeat-rich sex chromosome regions remains a huge challenge. Comparisons with other cichlids from LT will answer the question if LG5 has indeed more often and repeatedly evolved as a sex chromosome in cichlids and if the SD regions represent the co-option of an ancestral polymorphism. Extension to further species from LT will answer the question if this pattern is confined to the haplochromine lineage.

## Additional files

Additional file 1: Figure S1. Sampling location of the “Chipwa wild-caught” fish parents used in this study. (PNG 2140 kb)
Additional file 2: Table S1. Loci with male-specific coverage and higher coverage in daughters of the *A. burtoni* lab strain cross with corresponding location in BLAST on *O. niloticus* genome and PCR conditions and results for male specific loci. (XLSX 1227 kb)
Additional file 3: Table S2. RAD SNPs differentiating males and females of the *A. burtoni* lab strain with corresponding BLAST results on the *O. niloticus* and *A. burtoni* genome. SNPs in bold show inheritance of alleles from father to sons only, supporting an XX-XY system. (XLS 56 kb)
Additional file 4: Table S3. RAD loci differentiating males and females of the *A. burtoni* Chipwa wild-caught fish with corresponding BLAST results on the *O. niloticus* genome. SNPs in bold show inheritance of alleles from father to sons only, supporting an XX-XY system. (XLS 807 kb)
Additional file 5: Table S4. Loci with male-specific coverage and higher coverage in daughters of the *A. burtoni* Chipwa wild-caught cross with corresponding location in BLAST on *O. niloticus* genome. (XLSX 83 kb)
Additional file 6: Table S5. GO annotations of *O. niloticus* genes located in the potential sex-determining regions on LG5, LG14 and LG18. (XLSX 64 kb)
Additional file 7: Figure S2. Gene expression of *A. burtoni* lab strain along the reference chromosomes of the Nile tilapia. Gene expression in male and female gonads of the *A. burtoni* lab strain along the reference chromosomes of the Nile tilapia as in Fig. 3c. (ZIP 329 kb)
Additional file 8: Table S6. Gene expression details of genes with strongest male and female bias on LGs 5 and 14 as well as gene candidates on LG5. (XLSX 30 kb)

## Abbreviations

ESD: Environmental sex determination
GO: Gene ontology
GSD: Genetic sex determination
kb: Kilo bases
km: Kilo meters
LG: Linkage group
LM: Lake Malawi
LT: Lake Tanganyika
LV: Lake Victoria
MB: Mega bases
my: Million years
OB: Orange blotch
RAD sequencing: Restriction site associated DNA sequencing
SD: Sex determination
SNP: Single nucleotide polymorphism
