# Supplementary figures and images for: Variations on a theme: Genomics of sex determination in the cichlid fish Astatotilapia burtoni
Source: BMC Genomics. 2016 Nov 7;17:883. doi: 10.1186/s12864-016-3178-0 (PMC5100337; doi:10.1186/s12864-016-3178-0)

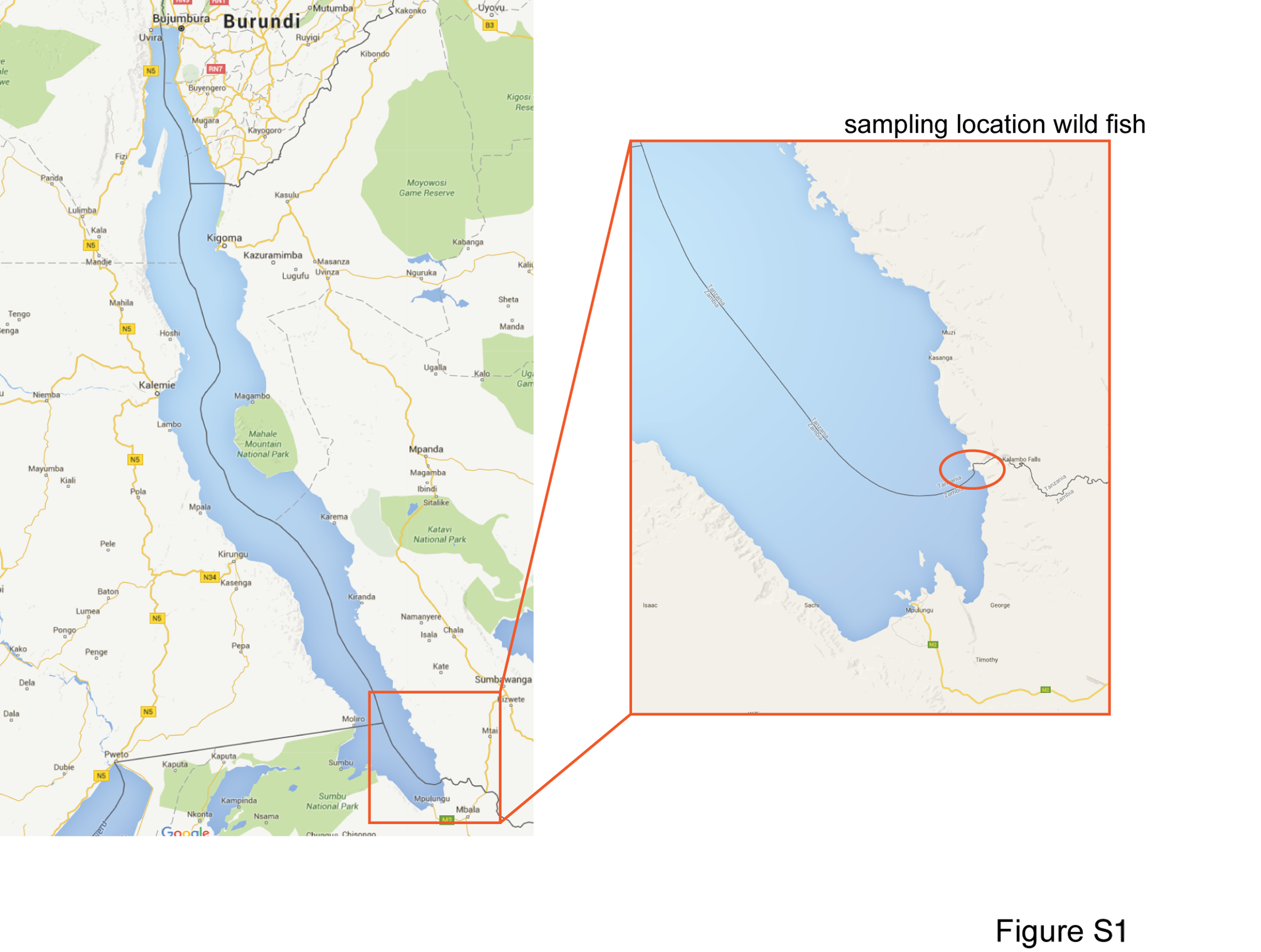

Supplement: Additional file 1: Figure S1. — Sampling location of the “Chipwa wild-caught” fish parents used in this study. (PNG 2140 kb) [file 12864_2016_3178_MOESM1_ESM.png]

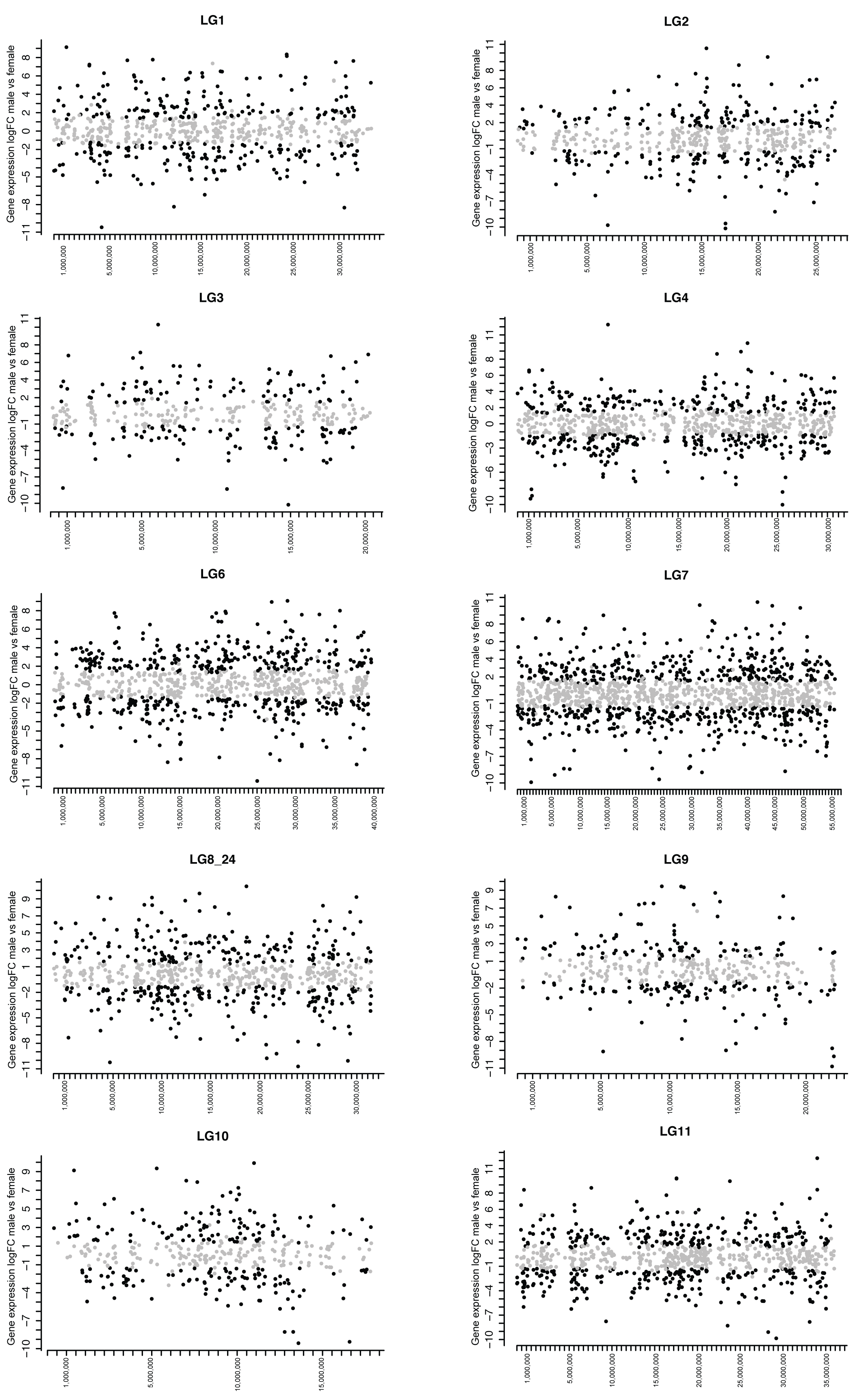

Supplement: Additional file 7: Figure S2. — Gene expression of A. burtoni lab strain along the reference chromosomes of the Nile tilapia. Gene expression in male and female gonads of the A. burtoni lab strain along the reference chromosomes of the Nile tilapia as in Fig. 3c. (ZIP 329 kb) [file 12864_2016_3178_MOESM7_ESM.zip › FigureS2a.png]

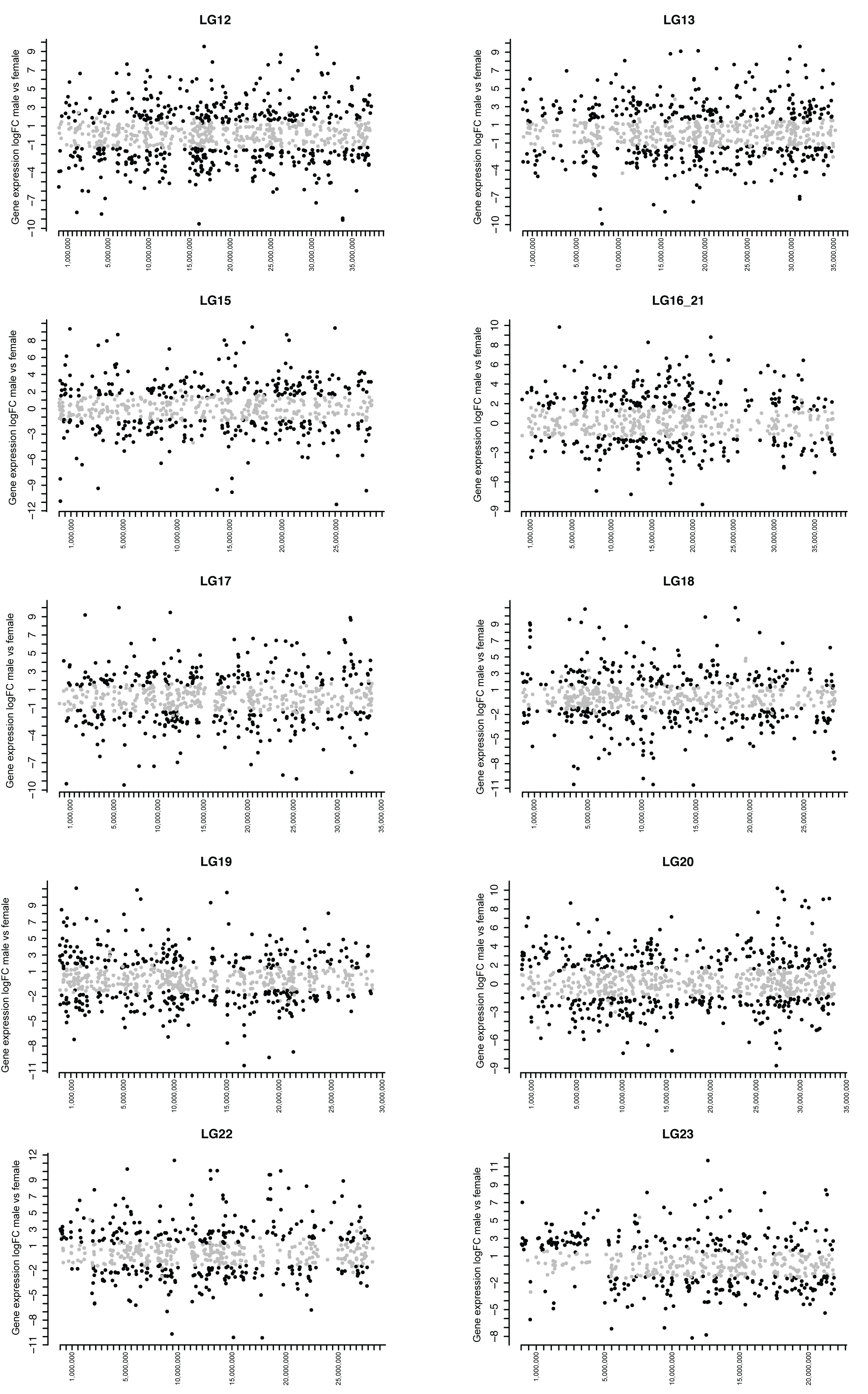

Supplement: Additional file 7: Figure S2. — Gene expression of A. burtoni lab strain along the reference chromosomes of the Nile tilapia. Gene expression in male and female gonads of the A. burtoni lab strain along the reference chromosomes of the Nile tilapia as in Fig. 3c. (ZIP 329 kb) [file 12864_2016_3178_MOESM7_ESM.zip › FigureS2b.png]
